# Facing Antimicrobial Resistance and Virulence in *Streptococcus agalactiae* During Late Pregnancy: Evaluation of *Lactobacilli* as a Supportive Approach

**DOI:** 10.1002/mbo3.70347

**Published:** 2026-06-23

**Authors:** Astri D. Tagueha, Giulia Radocchia, Daniela Scribano, Matteo Lo Scalzo, Giacinto Panella, Serena Schippa, Giovanni Gherardi, Roberta Creti, Ilaria Marani, Giovanna Alfarone, Monica Imperi, Massimiliano Marazzato, Lucia Nencioni, Paola Checconi, Dolores Limongi, Anna Teresa Palamara, Cecilia Ambrosi

**Affiliations:** ^1^ Department of Public Health and Infectious Diseases Sapienza University of Rome Rome Italy; ^2^ Department of Life Science, Health, and Health Professions Link Campus University Rome Italy; ^3^ Department of Human Sciences and Promotion of the Quality of Life San Raffaele Open University Rome Italy; ^4^ U.O.C Patologia Clinica, Ospedale Fabrizio Spaziani Frosinone Italy; ^5^ Department of Infectious Diseases Istituto Superiore di Sanità Rome Italy; ^6^ Department of Public Health and Infectious Diseases Sapienza University of Rome, Laboratory Affiliated to Institute Pasteur Italia‐Cenci Bolognetti Foundation Rome Italy; ^7^ Laboratory of Microbiology IRCCS San Raffaele Roma Rome Italy

**Keywords:** antibiotic‐resistance, Lactobacilli, pregnancy, *Streptococcus agalactiae*, virulence

## Abstract

*Streptococcus agalactiae* (Group B streptococcus, GBS) remains a major cause of neonatal sepsis and meningitis, despite the success of intrapartum antibiotic prophylaxis. However, the rising trend of antimicrobial resistance and the emergence of hypervirulent lineages highlight the need for complementary, innovative prophylactic strategies. This study aimed to characterize the resistance and virulence repertoire of colonizing GBS isolates and to assess the antagonistic potential of selected *Lactobacillus* strains under conditions mimicking the vaginal niche. We performed integrated phenotypic, genotypic, and functional analysis of 31 clinical GBS isolates collected during routine pre‐partum screening, focusing on antimicrobial susceptibility, hemolytic and aggregation phenotypes, capsular serotypes, pilus island profiles, and virulence genes. The collection included MDR isolates, strains exhibiting inducible clindamycin resistance and high‐level gentamicin resistance, as well as hypervirulent ST‐17 related lineages, with marked heterogeneity in β‐hemolytic activity and aggregation phenotypes linked to specific combinations of capsule, pilus, and virulence genes. In co‐culture experiments with HeLa cells, individual *Lactobacillus* strains exerted strong, strain‐dependent inhibition of GBS, preserving epithelial cell viability, and impairing pathogen adhesion by up to 98%. Importantly, the multi‐strain *Lactobacillus* combination provided broader and more consistent antagonistic activity, including against MDR, inducible clindamycin‐resistant, hypervirulent ST‐17‐associated, and strongly hemolytic GBS isolates. Although these findings are preliminary, being derived from a limited number of isolates and an in vitro model, they support the promising potential of rationally selected multi‐strain *Lactobacillus* formulations as a strategy to reduce colonization by high‐risk GBS lineages and warrant further investigation.

## Graphical abstract

Integrated phenotypic, genotypic, and functional characterization of clinical *Streptococcus agalactiae* (Group B streptococcus, GBS) isolates from maternal carriage. Combination of selected *Lactobacillus* species effectively antagonize *S. agalactiae* by limiting bacterial growth, preserving epithelial cell viability, and reducing pathogen adhesion, supporting their use as promising adjuncts to antibiotic intrapartum prophylaxis for the prevention of neonatal GBS disease.

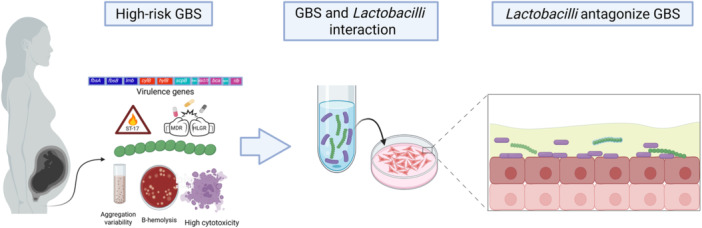

## 1 Introduction

*Streptococcus agalactiae* (Group B Streptococcus, GBS) are Gram‐positive, β‐hemolytic bacteria that represent one of the leading causes of neonatal sepsis, pneumonia, and meningitis worldwide, being associated with substantial mortality and long‐term neurodevelopment impairment (Puopolo et al. 2022; Paul et al. 2023; Li et al. 2025). Indeed, GBS can asymptomatically colonize the maternal gastrointestinal or genitourinary systems, thereby spreading from mother to child during labour and delivery (Furuta et al. 2022). It was reported that in 2020, 19.7 million pregnant women were positive for GBS in the recto‐vaginal region, determining an estimated 58,300 infant deaths, 46,200 stillbirths, and 518,000 GBS‐associated preterm births, globally (Gonçalves et al. 2022). Furthermore, 390,000 infants developed early‐ or late‐onset GBS disease, with around 40,000 survivors affected by long‐term neurological sequelae (Paul et al. 2023). GBS harbors several virulence factors that enhance its ability to colonize mucosal surfaces, adhere to host tissues, evade immune defenses, allowing the development of invasive GBS disease. These include genes encoding proteins involved in adhesion (*fbsA/B*, and *lmb*), in hemolysis (*cylB* and *hylB*), in immune evasion (*scpB*, and *bac*), and surface‐anchored alpha‐like proteins (*alpha‐C* or *bca*, *epsilon or alp1*, *alp2*/3, and *alp4*). Together, these virulence factors drive the colonization capacity and invasive potential of clinical strains (Bobadilla et al. 2021; EL‐Lakany et al. 2023; Zeng et al. 2024). Notably, hypervirulent strains, carrying specific surface factors, such as HvgA and Srr2, are strongly associated with neonatal meningitis; some GBS lineages also developed high‐level gentamicin resistance (HLGR), an antibiotic widely used as therapy in neonatal sepsis and meningitis (Creti et al. 2022). Many of these hypervirulent isolates belong to the clonal complex 17 (CC17), particularly sequence type 17 (ST17), a lineage widely recognized as highly adapted to neonatal infection and associated with increased invasive potential and central nervous system tropism (Lamy et al. 2006; Hsu et al. 2023). In the USA and several European countries, recto‐vaginal screening at 35–37 weeks of gestation was introduced to reduce the burden of neonatal GBS infections, followed by intrapartum antibiotic prophylaxis (IAP) for colonized mothers (Panneflek et al. 2024; Lohrmann et al. 2025). While this strategy has significantly reduced the incidence of neonatal early‐onset sepsis, it may be a major driver for the general increase in antibiotic resistance among GBS strains and does not guarantee prevention of GBS‐related abortions, stillbirths, preterm births, and late‐onset disease (Bostanghadiri et al. 2025; Hsu et al. 2025). In addition, extensive IAP exposure may hamper the establishment and development of the neonatal microbiota, with potential long‐term health implications, including the predominance of pathogenic bacteria (Nogacka et al. 2017; Stearns et al. 2017; Ainonen et al. 2022; Iqbal et al. 2025). In light of these concerns, there is growing interest in identifying alternative or complementary strategies to control maternal GBS colonization during pregnancy. Among the most promising candidates are probiotic *Lactobacillus* spp. strains, which may exert antimicrobial effects through competitive exclusion, production of inhibitory substances, and modulation of the local immune response (Ruíz et al. 2012; Ortiz et al. 2014; Martín et al. 2019; Marziali et al. 2019). Thus, the aim of this study was to characterize a collection of GBS clinical isolates, by assessing their antimicrobial susceptibility, hemolytic activity, aggregation capacity, and virulence gene profiles, while also evaluating the ability of selected commercial *Lactobacillus* spp. strains to inhibit GBS growth, both under co‐culture conditions and in an in vitro cervical epithelial cell adhesion model. Being hemolytic activity and aggregation behavior associated with colonization efficiency and pathogenic potential, their evaluation allowed us to investigate whether isolates differed in traits that may influence host‐pathogen interactions and, consequently, the effectiveness of probiotic interference strategies. Our findings demonstrated the strong inhibitory potential of combined *Lactobacillus* spp. strains against currently circulating GBS isolates, supporting the further development of strain‐specific probiotic approaches as alternative or complementary options to current antibiotic protocols.

## 2 Materials and Methods

### 2.1 GBS Collection

Thirty‐one clinical GBS isolates were collected between January and March 2025 as part of a routine screening for pathogen colonization of pregnant women at the end of the third trimester of gestation (35–37 weeks), from uro‐vaginal‐rectal swabs (*n* = 26), and urine (*n* = 5). No additional selection criteria were applied, and all GBS isolates recovered during the study period were included in the analysis. Species identification was conducted using matrix‐assisted laser desorption ionization‐time of flight mass spectrometry (MALDI‐TOF MS). Samples were preserved at −80°C in glycerol stocks in Todd Hewitt broth. For experiments, isolates were routinely cultured in Brain Heart Infusion (BHI) medium, unless differently specified.

### 2.2 Antibiogram Profiles

The antimicrobial susceptibility profiles of GBS were determined using the VITEK2 system (bioMérieux, Italia S.p.A, Grassina, Italy). Interpretation was performed according to the European Committee on Antimicrobial Susceptibility Testing (EUCAST) guidelines (v14.0, 2024), using the corresponding minimum inhibitory concentration (MIC) breakpoints for each antibiotic class (www.eucast.org). For specific antibiotics, such as tetracycline, clindamycin, and erythromycin, susceptibilities were initially assessed by MIC and then verified with disk diffusion tests. The detection of inducible clindamycin resistance was performed using the D‐test method, according to the EUCAST guidelines, as previously described (Imperi et al. 2024). Erythromycin resistance phenotypes were classified as inducible (IR), constitutive (CR), and clindamycin‐susceptible isolate (M phenotype), and fully susceptible (S), as previously described (Kamińska et al. 2024). HLGR was assessed at the recommended concentration (500 µg/mL). The presence of associated antibiotic resistance genes (*ermA, ermB, mef, tet(M), tet(O)*, and *aac(6’)‐Ie‐aph(2”)‐Ia* was assessed as previously described (Vakulenko et al. 2003; Imperi et al. 2010; Creti et al. 2021). MDR was defined as resistance to at least three different classes of antibiotics (Magiorakos et al. 2012).

### 2.3 Hemolytic Test

A single colony of each GBS clinical isolate from a freshly streaked BHI agar plate was inoculated into 3 mL of BHI broth and incubated at 37°C for 18–24 h under aerobic conditions. Subsequently, 10 µL of the culture were spotted onto Columbia blood agar plates (Becton Dickinson, Milan, Italy) and incubated under identical conditions. Following incubation, the bacterial spots were inspected to determine the presence and type of hemolysis. The β‐hemolytic phenotype was classified into four categories: non‐haemolytic; weak, characterized by a narrow, faint, or poorly defined zone of clearance; moderate, exhibiting a medium‐sized zone with less distinct edges and a yellowish translucency under transmitted light; and strong, displaying a clear and transparent zone.

### 2.4 Aggregation Test

The ability of GBS to auto‐aggregate was assessed, as previously described (Marziali et al. 2019), with minor modifications. In brief, overnight cultures were harvested by centrifugation at 10,000*g* for 10 min, washed twice with phosphate‐buffered saline (PBS), and resuspended in PBS to an initial OD_600_ (referred to as A_0_) of 0.5. The bacterial suspensions were then incubated overnight at room temperature without agitation, and the OD_600_ (A_t_) was measured. The percentage of auto‐aggregation was calculated according to the following formula:

$$\mathrm{Aggregation}\;\left(\%\right)=1-\frac{A_{t}}{A_{0}}x\;100$$

The aggregation percentage was then converted to z‐scores to define aggregation categories to avoids arbitrary percentage cut‐offs, using following formula:

$$z\;\mathrm{score}=\frac{\mathrm{Aggregation}-\mathrm{mean}\;\mathrm{all}\;\mathrm{values}}{\mathrm{standard}\;\mathrm{deviation}}$$

Based on *z* score, GBS were further classified into four categories: non‐aggregating (z ≤ −1), weak aggregating (−1 < z < 0), moderate aggregating (0 ≤ z < 1), and strong aggregating (z ≥ 1).

### 2.5 Serotyping, Detection of Pilus Island, Virulence Genes, and Hypervirulent ST‐17

Serotyping of GBS was performed using the commercial latex agglutination test ImmuLex StrepB‐Kit (SSI Diagnostica, Hillerød, Denmark) (Afshar et al. 2011; Slotved and Hoffmann 2017). Molecular typing by multiplex‐Polymerase Chain Reaction (PCR) was further applied in case of not‐typeable strains and to confirm the results of the agglutination test, as previously reported (Imperi et al. 2010). The presence of pilus islands (PI‐1, PI‐2a, and PI‐2b) and virulence genes (*fbsA, fbsB, lmb, cylB, hylB, scpB, bac, alp2/3, alp4, alpha‐C*, epsilon, and *rib)* was detected by multiplex PCR, as previously described (Creti et al. 2004; Springman et al. 2014; Bobadilla et al. 2021). In addition, a singleplex PCR was used to detect the *hvgA* gene as a marker of the hypervirulent ST‐17 lineage (Lamy et al. 2006). The resulting amplicons were separated on 2% agarose gels stained with SafeView Classic (abm, Canada) and visualized using a ChemiDoc Imaging System (Bio‐Rad, USA). All the primers used in this study are listed in Supporting Information S1: Table S3.

### 2.6 Evaluation of Antagonistic Activity of *Lactobacilli* Against GBS

Four *Lactobacillus* spp. strains belonging to four different species, *Lactobacillus plantarum* strain NCIMB 30437, *Lactobacillus paracasei* strain NCIMB 30439, *Lactobacillus rhamnosus* strain CRL1505 (DSM 29673), and *Lactobacillus reuteri* strain NCIMB 30242 (referred to as *L. plantarum*, *L. paracasei*, *L. rhamnosus*, and *L. reuteri* throughout the whole text) were selected for this study. The inhibitory activity of the *Lactobacillus* spp. strains against clinical GBS isolates was first qualitatively evaluated using a co‐plating assay and then quantified through co‐culture experiments in BHI broth. For the co‐plating assay, single colonies of each *Lactobacillus* and GBS strain were individually inoculated into BHI broth and incubated aerobically at 37°C for 24 h. Next, 150 µL of each *Lactobacillus* culture was spread onto BHI agar, and 10 µL of each GBS culture was spotted onto the surface. Plates were incubated at 37°C for 24 h. Growth inhibition, defined as the presence of a clear zone around the GBS spots, was interpreted as a positive for antagonistic activity. GBS isolates susceptible to *Lactobacillus* inhibition were selected for further co‐culture experiments. For the co‐culture assay, a freshly streaked single colony of each *Lactobacillus* strain and GBS isolate were co‐inoculated into 3 mL of BHI broth and incubated under the same conditions; corresponding monocultures were included as growth controls. Following incubation, co‐culture and monoculture suspensions were serially diluted in PBS and plated onto MRS agar (for *Lactobacilli*) and BHI agar (for GBS) to determine colony‐forming units (CFUs)/ml. GBS colonies were enumerated after 24 h, whereas *Lactobacillus* were enumerated after 48 h to allow sufficient growth on solid medium. The mortality rate and competition index (CI) were calculated using the following formulas:

$$\text{Mortality rate}\;=\frac{\text{CFU monoculture}\;-\;\text{CFU co}-\;\text{culture}}{\text{CFU monoculture}}\times 100\%$$

$$\text{Competition index}\;=\frac{\left(\text{CFU}\;\mathrm{Lactobacilli}\text{co}-\text{culture}/\text{CFU}\;S.\;\mathrm{agalactiae}\text{co}-\text{culture}\right)}{\left(\text{CFU}\;\mathrm{Lactobacilli}\text{monoculture}/\text{CFU}\;S.\;\mathrm{agalactiae}\text{monoculture}\right)}$$

### 2.7 Cytotoxicity of *Lactobacillus* spp. and GBS Isolates on Host Cells

The cytotoxicity of selected *Lactobacillus* spp. strains and of GBS was individually evaluated on human cervical carcinoma HeLa (CCL‐2) cell monolayers cultured in Dulbecco's Modified Eagle Medium (DMEM) supplemented with 10% fetal bovine serum (FBS), 1% glutamine, 1% penicillin‐streptomycin, and 50 μg/mL gentamicin. HeLa cells (2.5 × 10^5^ per well) were seeded onto 24‐well plates (Corning®) and incubated overnight to reach > 70% confluency. *Lactobacillus* cultures were harvested by centrifugation at 4000*g* for 15 min and resuspended in antibiotic‐free DMEM containing 5% FBS. After washing with PBS, HeLa cell monolayers were exposed either to individual *Lactobacillus* spp. strains or to a mixture of the four strains, using a multiplicity of infection (MOI) of 1000. Cells were incubated at 37°C with 5% CO_2_ for 1.5 and 3 h. After five washes with PBS, cytotoxicity was assessed using an MTT assay, as previously reported (Scribano et al. 2020). Briefly, 300 µL of the MTT solution (0.5 mg/mL in DMEM without phenol red; Sigma‐Aldrich) was added to each well and allowed to incubate for 3 h. Subsequently, 500 µL of 0.1 N HCl in isopropanol was added to solubilize the formazan crystals, and plates were incubated for 1 h before measuring absorbance at 570 nm using a microplate reader (BioTek). To evaluate the combined cytotoxic effects of GBS and *Lactobacillus* spp. strains, HeLa cell monolayers were first infected with GBS at an MOI of 10 for 30 min. Then, the medium was removed and replaced with the mixture of *Lactobacillus* at an MOI of 1000, followed by incubation for 1.5 or 3 h before performing the MTT assay. Untreated cells were used as controls. Cell viability was calculated with the following formula:

$$\mathrm{Viability}\;\left(\%\right)=\frac{{\mathrm{Mean}\;\mathrm{OD}}_{\mathrm{treated}}}{{\mathrm{Mean}\;\mathrm{OD}}_{\mathrm{control}}}\;x\;100\%$$

Optical microscopic images were acquired using a 20× objective on an Optika microscope (Optika Microscopes, Italy) equipped with a Tucsen MIchrome 20 camera.

### 2.8 Lactobacilli Host Cell Protection Assay Against GBS

To assess the ability of *Lactobacillus* spp. strains to displace adherent GBS isolates, HeLa cell monolayers were first infected with GBS and then exposed to the mixture of the four *Lactobacillus* spp. strains, as described above. After 3 h of incubation, cell monolayers were extensively washed with PBS to remove loosely adherent bacteria and then lysed with 1 mL of 0.1% Triton X‐100 to recover adherent bacteria. Lysates were serially diluted and plated onto BHI (for GBS) and MRS agar (for *Lactobacillus* spp.) to determine colony‐forming units (CFU)/mL. Untreated cells, as well as cells individually infected with GBS, or *Lactobacillus* spp. strains were included as controls. The percentage inhibition index of GBS was calculated using the following formula:

$$\%\text{Inhibition}=1-\frac{\text{CFU co}-\text{culture}}{\text{CFU monoculture}}\times 100\%$$

Optical microscopic images of methanol‐fixed, Giemsa‐stained monolayers were acquired as described above.

### 2.9 Statistical Analysis

Statistical analyses were performed using GraphPad Prism Software (version 11). Continuous data from aggregation assays are presented as z‐scores. For the MTT assay, data were first tested for normality using the Shapiro‐Wilk test, and homogeneity of variance was assessed by inspection of residual plots. Under these assumptions, differences in cell viability among multiple treatment groups were analyzed using one‐way ANOVA and followed by Dunnett's post hoc test to compare each treatment group to the control. Differences in bacterial adherence on HeLa cells between treated cells and control conditions were assessed using two‐tailed paired *t*‐test after confirmation of approximate normality of the paired differences. Associations between genotypic and phenotypic characteristics were evaluated using Spearman's rank correlation. *p* values < 0.05 were taken as being statistically significant. Data from MTT assay and from each experiment sets were first tested for normality using the Shapiro–Wilk test, and homogeneity of variance was assessed by inspection of residual plots.

## 3 Results

### 3.1 Emerging Rifampicin Resistant GBS Isolates

All GBS isolates were susceptible to penicillin, linezolid, moxifloxacin, teicoplanin, tigecycline, trimethoprim/sulfamethoxazole, and vancomycin (Supporting Information S1: Table S1). In contrast, resistance was observed to tetracycline (74.2%, 23/31), rifampicin (71%, 22/31), erythromycin (25.8%, 8/31), clindamycin (22.6%, 7/31), and levofloxacin (3.2%, 1/31) (Supporting Information S1: Table S1). Five isolates were erythromycin‐clindamycin‐resistant (CR phenotype), including four serotype V and one of serotype III, and harbored the *ermB* gene (16%, 5/31) (Table 1 and Supporting Information S1: Table S1). Two erythromycin‐resistant isolates with inducible IR phenotype were detected: one serotype Ia isolate carrying the *ermA* gene, while the other was a serotype II negative for all tested resistance genes (Table 1). In addition, one erythromycin‐resistant and clindamycin‐susceptible isolate (M phenotype) of serotype Ia carried the *mef* gene (Table 1). All erythromycin‐ and clindamycin‐susceptible isolates (S phenotype, 74.2%, 23/31) lacked resistance genes (Table 1 and Supporting Information S1: Table S2). Among 23 tetracycline‐resistant isolates, all carried at least one resistance gene, with *tetM* being the most prevalent (82.6%, 19/23), followed by *tetO* (17.4%, 4/23). MDR phenotype, defined by the resistance to tetracycline‐erythromycin‐clindamycin, was observed in 22.6% (7/31) of isolates with 57.1% (4/7) additionally resistant to rifampicin (Table 1 and Supporting Information S1: Table S1). MDR isolates belonged to serotype V (four isolates), Ia, II, and III (one isolate each) (Table 1). Overall, three isolates (9.7%), two of serotype IV and one serotype II, exhibited HLGR and carried the *aac(6’)‐Ie‐aph(2”)‐Ia* gene (Table 1 and Supporting Information S1: Table S2).

**Table 1:** Microbiological characteristics of 31 studied GBS clinical isolates.

Serotype (n. isolates)	Alpha‐like protein genes (n. isolates)	Pilus island (PI) (n. isolates)	*hvg*A gene (ST‐17) (n. isolates)	Erythromycin phenotype/genotype (n. isolates)	Tetracyline phenotype/genotype (n. isolates)	HLGR^a^, n. isolates	MDR^c^
Ia (2)	epsilon (1), alp2/3 (1)	PI‐2a (2)	neg (2)	IR/*erm*A (1), M/*mef*A (1)	R/*tet*M (2)	0	1
II (6)	rib (5), neg (1)^b^	PI‐1 + 2a (3), PI‐2a (3)	neg (6)	IR/neg (1)^b^, S (5)	R/*tet*M (3), R/*tet*O (2), S (1)	1	1
III (11)	rib (11)	PI‐1 + 2b (8), PI‐1 + 2a (2), PI‐2b (1)	pos (9), neg (2)	CR/*erm*B (1), S (10)	R/*tet*M (7), R/*tet*O (2), S (2)	0	1
IV (4)	alpha‐C (3), rib (1)	PI‐1 + 2a (2), PI‐2b (2)	pos (3), neg (1)	S (4)	R/*tet*M (1), S (3)	2	0
V (6)	alp2/3 (4), alpha‐C (2)	PI‐1 + 2a (4), PI‐2a (2)	neg (6)	CR/*erm*B (4), S (2)	R/*tet*M (6)	0	4
VI (2)	alpha‐C (2)	PI‐1 + 2a (2)	neg (2)	S (2)	S (2)	0	0

*Note:* ^a^All HLGR isolates carried the *aac(6’)‐Ie‐aph(2”)‐Ia* resistance gene. ^b^One serotype II isolate was negative for all alpha‐like surface protein genes tested (*epsilon, rib, alpha‐C, alp2/3*, and *alp4*) and showed IR phenotype with no erythromycin resistance genes detected. ^c^MDR, multidrug‐resistant isolates, contemporarily resistant to at least 3 classes of antibiotics (erythromycin, clindamycin and tetracycline).

Abbreviations: CR, erythromycin resistance with constitutive clindamycin resistance; HLGR, high‐level gentamycin resistance; IR, erythromycin resistance with inducible clindamycin resistance; M, erythromycin resistance and clindamycin susceptibility; S, erythromycin and clindamycin susceptibility.

### 3.2 GBS Showed Different Hemolytic Patterns

**Figure 1:**
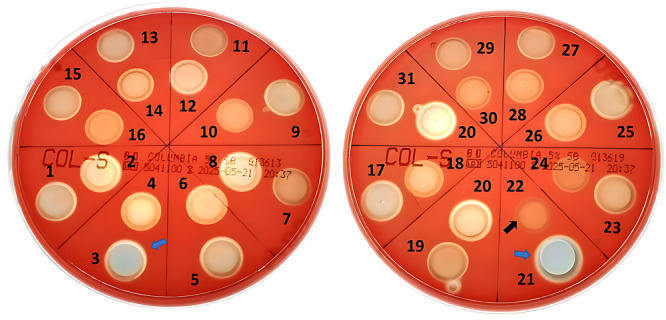
GBS hemolytic activity. Ten microliters of the overnight cultures were spotted onto Columbia blood agar plates and incubated for 18–24 h in aerobic condition. Strong β‐hemolytic isolates are indicated by blue arrows, whereas the γ‐hemolytic strain is indicated by a black arrow. Numbers represent strain IDs.

To classify the hemolytic properties of our GBS isolate collection, strains were spotted onto Columbia blood agar plates (Figure 1). Overall, the intensity and appearance of a β‐hemolytic varied among our GBS collection. Two isolates showed the strongest β‐hemolysis (6%, 2/31), 11 isolates exhibited moderate hemolysis (35%, 11/31), while the majority displayed a weak β‐hemolysis (55%, 17/31) (Figure 1 and Table 2). Notably, only isolate 22 showed no visible clearing zone, suggesting a γ ‐hemolytic phenotype (Figure 1).

**Table 2:** Hemolytic patterns of clinical GBS isolates.

Hemolysis category	Pattern	Strains
β	Strong	3, 21
	Moderate	1, 2, 4, 5, 6, 8, 9, 12, 17, 20, and 25
	Weak	7, 10, 11, 13, 14, 15, 16, 18, 19, 23, 24, 26, 27, 28, 29, 30, 31
γ	None	22

### 3.3 Variable Aggregation Capacity Among Clinical GBS

**Figure 2:**
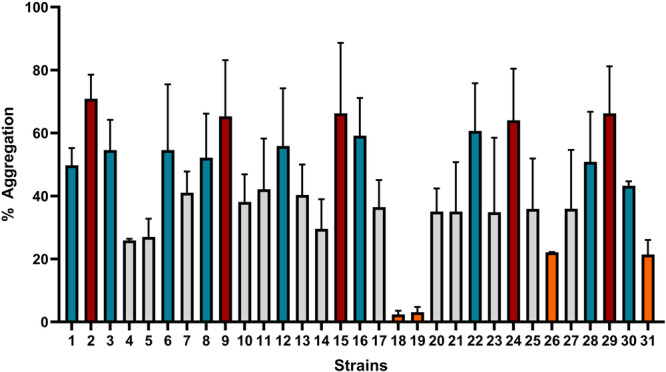
Aggregation ability of GBS clinical isolates. Aggregation profiles, expressed as the percentage of aggregated cells after overnight incubation. Bars represent the mean ± standard deviation (SD) from three independent experiments (*n* = 6). Based on z‐scores, isolates were classified into four categories, indicated by color codes: non‐aggregating (orange), weak (grey), moderate (green), and strong (red) aggregators.

Next, we evaluated the aggregation capacity across our GBS collection. Overall, isolates exhibited marked variability in aggregation, with 42% (13/31) classified as weak aggregators, 29% (9/31) as moderate, and 16% (5/31) as strong, while 13% (4/31) showed no detectable aggregation under the tested conditions (Figure 2).

### 3.4 Diverse Serotype and Pilus Island Profiles Among GBS Isolates

The serotype distribution and pilus island (PI) profiles were analyzed to characterize key determinants of GBS colonization and virulence. Among the 10 well‐characterized GBS serotypes, our clinical isolates belonged to six groups, with serotype III being the most prevalent (35.5%, 11/31), followed by serotype II and V (19.3% each, 6/31), and IV (12.9%, 4/31), and Ia and VI (6.4%, 2/31) (Table 1). Notably, the most prevalent serotypes were associated with diverse PI profiles. The PI‐1 + 2b combination was exclusively detected in the majority of serotype III (8/11, 72.7%). PI‐1 + 2a occurred in all serotypes but one, with the highest proportion observed in serotype V (4/6, 66.7%). The PI‐2a profile was detected in three serotypes, Ia, II and V. In contrast, the less frequent PI‐2b profile was restricted to serotype III and IV (Table 1). Collectively, these findings highlight the marked genetic diversity of surface structures within our clinical GBS collection.

### 3.5 GBS Showed a High Prevalence of Key Virulence Associated‐Genes

**Figure 3:**
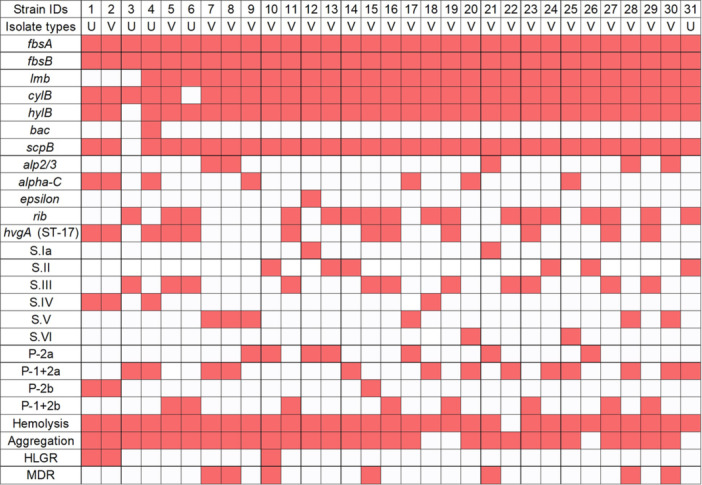
Distribution of virulence genes and phenotypic traits among GBS clinical isolates. The presence/absence of genes is indicated by filled and empty boxes, respectively. Isolate sources indicated by symbol, U for urine and V for vagina.

To assess the presence and distribution of virulence determinants, known virulence‐associated genes were analyzed using a combination of singleplex and multiplex PCR assays (Figure 3). The genes *fbsA, fbsB* and *lmb*, encoding adhesion‐related proteins involved in fibrinogen and laminin binding, were highly prevalent, being identified in 100% of isolates for *fbsA, fbsB*, and 90% (28/31) for *lmb*. The *cylB* and *hylB* genes, essential for cytolysin/hemolysin production, were detected in the vast majority of isolates (30/31, 97%), consistent with the predominant β‐hemolytic phenotype observed (Figures 1 and 3). The *scpB* gene, encoding the C5a peptidase involved in immune evasion, was identified in the entire strain collection but one (30/31, 97%), whereas the gene encoding the surface Cβ protein (*bac*), which binds IgA and complement factor H, was rare, and detected in only one strain (1/31, 3%). Among the genes encoding surface proteins, *rib* was the most prevalent (17/31, 54.8%), typically associated with serotypes III and II, followed by *alpha‐C* (7/31, 22%). *alp2/3* was frequent in serotype V (4/6, 66.7%), and *epsilon* (1/31, 3%) was only identified in serotype Ia, while the *alp4* gene was not detected in any strain (Table 1). Noteworthy, isolate 10 lacked any alpha‐protein‐like genes. The hypervirulence‐associated gene *hvgA*, highly specific for the ST‐17 lineage, was detected in 12 of 31 isolates (39%), and mainly associated with serotype III (9/12, 75%) and serotype IV (3/12, 25%) (Table 1 and Figure 3).

Interestingly, most *hvgA*‐positive isolates were recovered from vaginal specimens (9/12, 75%). All these isolates carried the adhesion and invasion genes *fbsA, fbsB, cylB*, and *scpB*, and many harbored *rib* genes. Nearly all *hvgA*‐positive isolates were β‐hemolytic and displayed an aggregation phenotype. The predominant PI profile among hypervirulent isolates was PI‐1 + 2b (8/12, 67%), followed by PI‐2b (3/12, 25%), and the PI‐1 + 2a (1/12, 8%) (Figure 3). Additionally, more than half of the *hvgA*‐positive isolates exhibited resistance to tetracycline and rifampicin (58%, 7/12 each), and two showed HLGR phenotype (17%, 2/12). Despite this mosaic resistance pattern, only one hypervirulent ST‐17 isolate displayed a MDR profile with a CR phenotype (Table 1 and Figure 3).

### 3.6 *Lactobacillus* spp. Exhibit Inhibitory Activity Against Clinical GBS Isolates

**Figure 4:**
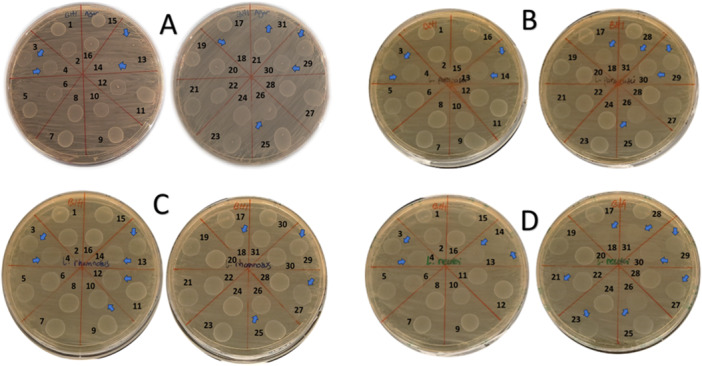
Inhibitory effects of *Lactobacillus* spp. on GBS clinical isolates. Plates were seeded with (A) *L. plantarum*, (B) *L. paracasei*, (C) *L. rhamnosus*, and (D) *L. reuteri* as a bacterial lawn, and 10 µL of all GBS isolates were spotted on top. Sensitive isolates showing partial‐to‐clear zone are indicated by blue arrows. Two independent experiments were performed in duplicate (*n* = 4). Representative images are shown.

Given the growing need of alternative or complementary strategies to limit GBS colonization, we set up a qualitative co‐plating assay to evaluate the antagonistic potential of selected *Lactobacillus* spp. strains against our GBS clinical isolates. Although the majority of GBS isolates were not markedly inhibited by selected *Lactobacillus* spp. strains, this initial screening revealed thin partial‐to‐clear areas surrounding the GBS spot zones, suggesting some degree of inhibition (Figure 4). In particular, 14 isolates were identified as species‐specific sensitive isolates (3, 4, 10, 12, 13, 14, 18, 21, 23, 26, 27, 29, 30, and 31). Among this subset of clinical isolates, 64% (9/14) were inhibited by *L. plantarum* and *L. paracasei*, 71% by *L. rhamnosus*, and 86% by *L. reuteri* (Figure 4).

**Figure 5:**
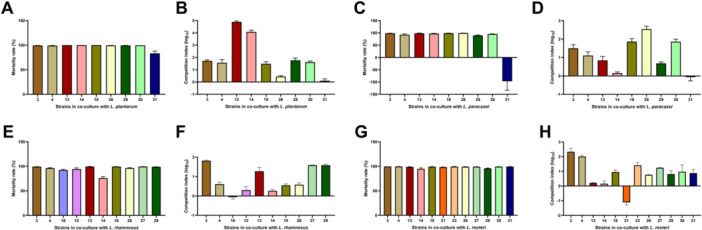
Inhibitory activity of *Lactobacillus* spp. against GBS in broth co‐cultures. GBS isolates previously identified as plate‐sensitive were tested in broth co‐culture assays with (A and B) *L. plantarum*, (C and D) *L. paracasei*, (E and F) *L. rhamnosus*, and (G and H) *L. reuteri*. Inhibitory effects were evaluated by determining GBS mortality rates (A, C, E, G), and by calculating the competition index (CI), expressed as log_10_‐transformed values (B, D, F, H). Based on log_10_CI values, GBS isolates were classified as non‐inhibited (log_10_CI < 0), weakly inhibited (0 < log_10_CI < 1), moderately inhibited (1 < log_10_CI < 2), or strongly inhibited (log_10_CI ≥ 2). Data are presented as mean ± SD from three independent experiments (*n* = 6). Only GBS isolates showing susceptibility in the co‐culture assay are reported.

To quantify the extent of GBS‐*Lactobacillus* growth inhibition, broth co‐culture assays were performed using different strain combinations (Figure 5). For most combinations, *Lactobacillus* spp. strains induced substantial killing of plate‐sensitive GBS isolates, with mortality values approaching 100% across all *Lactobacillus* strains (Figure 5A,C,E,G), including the three MDR isolates tested (strains 10, 21, and 30). In contrast, isolate 31 was the only one displaying a survival advantage when co‐cultured with *L. paracasei*, as indicated by a relative overgrowth of 1.92‐fold higher than those observed in its corresponding monoculture control (Figure 5C). Overall, these data indicate that, despite some strain‐specific differences, the *Lactobacillus* panel exerted a robust inhibitory effect on almost all tested GBS isolates.

To achieve a deeper understanding of the interaction between GBS isolates and *Lactobacillus* spp., the competition index (CI) was calculated by combining the survival outcomes of both species and expressing the results as log_10_ values. The CI profiles showed that *Lactobacillus* strains generally exerted moderate to strong inhibition of GBS isolates, although the interaction patterns were both isolate‐ and species/strain‐dependent (Figure 5B,D,F,H). Moderately to strongly inhibited GBS isolates accounted for 78% (7/9) in co‐colture with *L. plantarum*, and 67% (6/9) with *L. paracasei*, while *L. rhamnosus*, and *L. reuteri* showed inhibitory activity against 40% (4/10), and 33% (4/12) of GBS isolates, respectively. Weakly inhibited isolates were more frequent in co‐cultures with *L. rhamnosus* (5/10, 50%) and *L. reuteri* (5/12, 58%). Notably, non‐inhibited phenotypes (log_10_CI < 0) were clearly evident only for isolate 31 in combination with *L. paracasei*, consistent with the survival advantage previously observed for this pair (Figure 5C).

Noteworthy, some sensitive isolates, such as strains 10 and 21, displayed high mortality under co‐culture conditions (Figure 5E,G), but did not appear inhibited according to their CI values (Figure 5F,H). This apparent discrepancy can occur because the CI measures a relative ratio, and therefore it can hide high mortality if the surviving pathogen multiplies fast enough to balance or exceed *Lactobacillus* growth. In particular, isolate 21 antagonized *L. reuteri*, yielding a negative CI that reflects a relative overgrowth of pathogen (1.1‐fold higher) compared to the probiotic strain. In addition, the interaction between isolate 10 and *L. rhamnosus* resulted in a CI value close to zero, consistent with a balanced co‐culture in which neither species clearly dominates. These findings suggest that some GBS lineages may partially resist or even counteract the inhibitory potential of individual probiotic strains, as observed for isolates 10 and 21, both displaying an MDR phenotype.

### 3.7 *Lactobacillus* spp. Protect HeLa Cells From GBS‐Induced Cytotoxicity and Reduce Adhesion

**Figure 6:**
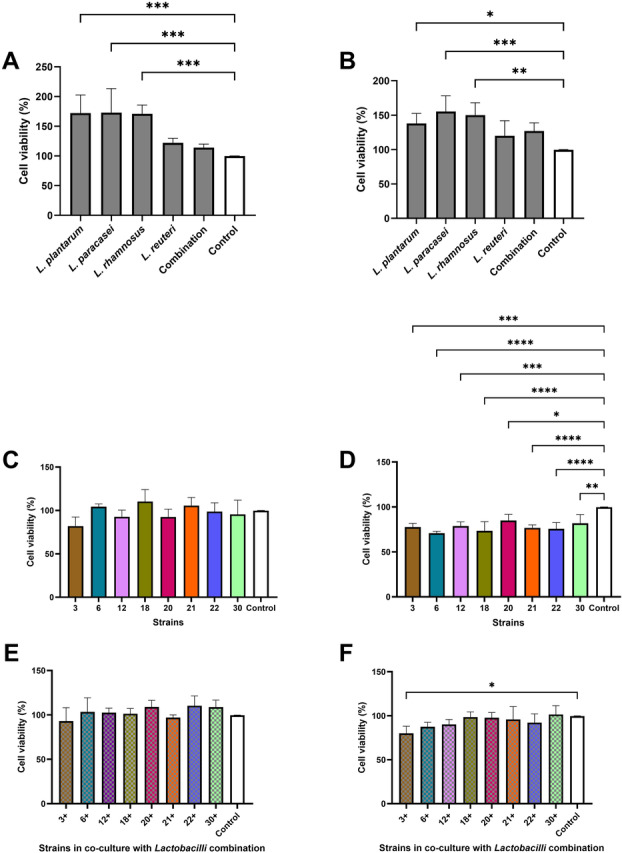
Viability of HeLa cells following exposure to *Lactobacillus* spp. strains, GBS, or their combination. Cell viability of confluent HeLa cell monolayers was determined at fixed time points using the MTT assay. (A and B) Exposure to individual *Lactobacillus* strains or to their four‐strain combination at an MOI of 1000 for 1.5 and 3 h, respectively. (C and D) Infection of selected GBS isolates at an MOI 10 for 1.5 and 3 h, respectively. (E and F) Sequential co‐infection (first with GBS isolate for 30 min and then with the *Lactobacillus* combination) at the indicated MOIs for 1.5 and 3 hpi, respectively. Data are expressed as percentages relative to uninfected control cells (mean ± SD from at least three independent experiments; *n* = 6). Statistical significance was assessed using one‐way ANOVA; *p < 0.05, **p < 0.01, ***p < 0.001, ****p < 0.0001.

To assess the safety of *Lactobacillus* spp. strains on human cells at high dosage, their cytotoxicity, either individually or as a combination, was evaluated using an MTT assay on HeLa cell monolayers exposed to an MOI of 1000 for 1.5 and 3 h (Figure 6). After 1.5 h of exposure, HeLa cell viability was equal to or higher than that of control cells, with significant increases observed for cells treated with *L. plantarum*, *L. paracasei*, and *L. rhamnosus* (Figure 6A). This beneficial profile persisted at 3 h, as all *Lactobacillus* strains preserved cell viability. Only cells exposed to *L. reuteri* alone or to the *Lactobacillus* combination showed a small further increase in cell viability compared with 1.5 h, although this difference did not reach statistical significance (Figure 6B). Next, the cytotoxicity potential of GBS isolates on HeLa cell monolayers was assessed, using an MOI of 10 for 1.5 and 3 h (Figure 6C,D). A panel of GBS strains (3, 6, 12, 18, 20, 21, 22, and 30) was chosen to represent different characteristics, including *Lactobacillus*‐sensitive and ‐resistant isolates, MDR and non‐MDR profiles, CR/IR/M phenotypes, hemolytic and non‐hemolytic isolates, aggregating and non‐aggregating phenotypes, carrying diverse alpha‐like protein genes, PI profiles, and the ST‐17 lineage. Interestingly, the impact of GBS on cell viability was both strain‐ and time‐dependent (Figure 6C,D). Indeed, after 1.5 h of infection, all strains induced only minimal and non‐significant reductions in cell viability, accounting for less than 5% (Figure 6C). Conversely, after 3 h of infection, GBS isolates caused a statistically significant decrease in cell viability, ranging from approximately 71% to 85% compared with the control cells (Figure 6D). Finally, to assess the protective role of *Lactobacillus* spp. strains in counteracting GBS‐induced cytotoxicity and preventing pathogen adhesion to epithelial cells, a co‐infection assay was performed. HeLa cell monolayers were first infected with representative GBS isolates at an MOI of 10 for 30 min, followed by the addition of the *Lactobacillus* combination at an MOI of 1000 for 1.5 and 3 h (Figure 6E,F). No differences in cell viability were observed at 1.5 h post‐infection (hpi) (Figure 6E). Conversely, at 3 hpi, cell viability remained above 90% for all GBS isolates tested, with the exception of strain 3, which reduced cell viability to values slightly but significantly below the control cells (Figure 6F).

**Figure 7:**
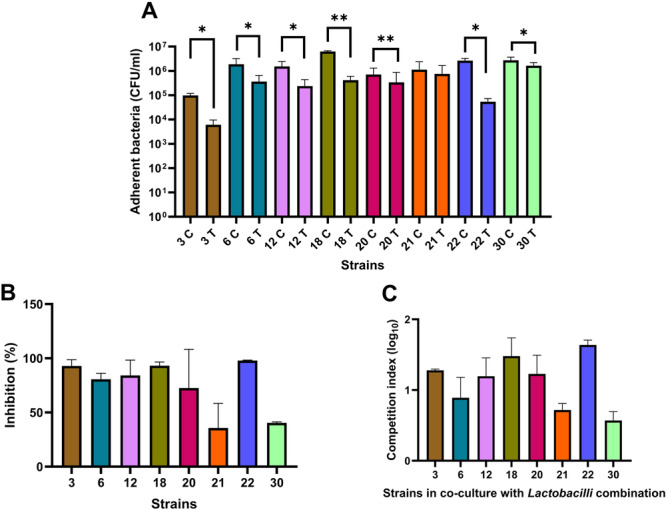
*Lactobacillus* combination‐mediated inhibition of GBS adherence to HeLa cells. Confluent HeLa cell monolayers were first infected with representative GBS isolates at an MOI of 10 for 30 min, followed by the addition of the *Lactobacillus* combination at an MOI of 1000 for 3 h. (A) Number of adherent GBS cells recovered from HeLa cell monolayers in the absence (C, controls) or presence (T, treated) of the *Lactobacillus* combination, expressed as CFU/mL. (B) Percentage of inhibition of GBS adherence, calculated from CFU counts. (C) Corresponding CI values. Data are presented as mean ± SD from at least three independent experiments (*n* = 6). Statistical significance of adherent bacteria was assessed using two‐tailed paired *t*‐test; *p < 0.05, **p < 0.01.

The same experimental setting was used to quantify the ability of the *Lactobacillus* strain combination to reduce/inhibit the adhesion of GBS isolates at 3 hpi (Figure 7), corresponding to the time point showing maximal cytotoxicity (Figure 6D). The presence of the *Lactobacillus* combination significantly reduced host cell adhesion for almost all GBS isolates, with inhibition values ranging from approximately 0.2‐ to 1.7‐fold relative to the corresponding control without *Lactobacillus* (Figures 7A,B). The strongest effects were observed for isolates 22, 18, and 3, which showed inhibition levels of about 98%, 93%, and 93%, respectively (Figure 7B). A weaker inhibition was observed for isolate 30, accounting for approximately 40%, whereas isolate 21 showed a non‐statistically significant reduction of 36% (Figure 7B). CI analysis further highlighted these differences (Figure 7C), ranking the extent of adhesion inhibition as follows: 22 > 18 > 3 > 12 > 6 > 20 > 30 > 21 (Figure 7C).

## 4 Discussion

GBS is still a leading cause of neonatal sepsis and meningitis worldwide, representing a major public health concern (Puopolo et al. 2022; Paul et al. 2023; Li et al. 2025). The implementation of universal screening programs and IAP has significantly reduced the incidence of early‐onset disease and its associated mortality (Russell et al. 2017; Panneflek et al. 2024; Oliveira et al. 2025). However, the increasing prevalence of MDR resistance strongly highlights the need to develop alternative and/or supportive therapeutic approaches, while awaiting an effective vaccine for maternal immunization (Gonçalves et al. 2022; Bostanghadiri et al. 2025; Hsu et al. 2025; Thorn et al. 2025). In our collection, two distinct resistance patterns were identified among the seven MDR isolates: rifampicin‐tetracyclines‐erythromycin‐clindamycin or tetracyclines‐erythromycin‐clindamycin. Notably, the rifampicin resistant was unexpectedly high, given that this antibiotic is not a first‐line agent for GBS. Rifampicin has previously been reported to be ineffective as GBS colonization treatment (Fernandez et al. 2001). In the absence of prior reports of rifampicin‐resistant GBS isolates from Italy, this finding needs further confirmation, including correlation with rifampicin use and genomic assessment of *rpoB* mutation (Patel 2023). Moreover, the strong alignment between disk diffusion test results and resistance genes, including the linkage between HLGR and *aac(6’)‐Ie‐aph(2”)‐Ia*, supports integration of phenotypic and genotypic data to obtain a more robust definition of resistance (Creti et al. 2021; Zakerifar et al. 2023). The presence of an MDR strain exhibiting HLGR highlights the risk of co‐selection with of gentamicin resistance with other determinants, which may compromise standard ampicillin‐gentamicin regimens for neonatal sepsis and meningitis management (McGee et al. 2021; Creti et al. 2022; Gong et al. 2024; Bostanghadiri et al. 2025).

Beyond antimicrobial resistance, the β‐hemolysin/cytolysin is a key virulence factor of GBS that triggers inflammation, disrupts host barriers, and promotes systemic dissemination, ultimately leading to severe neonatal infections (Sagar et al. 2013; Whidbey et al. 2013). In our GBS collection, most isolates displayed a β‐hemolytic phenotype, but with varying halo intensities from weak to moderate hemolysis, more frequently observed among vaginal isolates (*r* = −0.36, *p* = 0.045) (Supporting Information S1: Figure S1). Hemolysis heterogeneity has also been reported among human pathogenic strains (Mosabi et al. 1997; Lupo et al. 2014) and could be related to mutations within the *cyl* operon, which is essential for the biosynthesis, transport, and secretion of both β‐hemolysin and pigment production (Nizet 2002; Rosa‐Fraile et al. 2014). These phenotypic traits are closely interconnected, as non‐hemolytic strains are typically non‐pigmented; additionally, shifts in regulatory or environmental conditions may modulate their expression levels, ultimately varying the hemolytic halo intensity from strong to moderate or weak (Whidbey et al. 2013; Rosa‐Fraile et al. 2014). Moreover, the small proportion of γ‐hemolytic isolates observed in our study is consistent with a previous report describing this phenotype as infrequent, but still recognized among GBS clinical isolates (Zastempowska 2022). Notably, our γ‐hemolytic isolate carried both *cylB* and *hylB*; thus, the absence of hemolytic activity may be related to altered gene expression or additional regulatory or structural defects rather than the absence of hemolysin‐associated genes (Mosabi et al. 1997; Lupo et al. 2014).

Bacterial aggregation in static conditions reflects an initial step of microcolony formation, preceding biofilm formation, since this allows GBS cells to progressively settle and form thick, adhesive matrix over time (Miranda et al. 2018). The varying intensities of aggregation observed in our clinical isolates could be associated with strain‐specific differences in surface proteins and regulatory networks, as supported by the intermediate positive correlation detected between aggregation and PI‐2b (*r* = 0.412, *p* = 0.021, Supporting Information S1: Figure S1), and in agreement with previous evidence suggesting that aggregation is primarily linked to the expression of a key adhesin (Pietrocola et al. 2005; Konto‐Ghiorghi et al. 2009; Thomas and Cook 2022).

The predominance of serotype III across all PI profiles in our clinical collection, and the strong positive correlation with PI‐1 + 2b (*r* = 0.80, *p* < 0.0001, Supporting Information S1: Figure S1) align with previous Italian and international studies on neonatal disease and maternal colonization (Creti et al. 2021, 2025; EL‐Lakany et al. 2023; Lohrmann et al. 2025). Interestingly, we also found serotypes Ia and VI (6% each), the latter being rarely reported in humans and sharing genetic traits with isolates retrieved from animals (He et al. 2017). Two out of four serotype IV strains were HLGR and *hvgA*‐positive, reflecting characteristics previously reported among clinical GBS isolates (Creti et al. 2024). The serotype‐pilus association suggests that specific capsule‐pilus genotypes may confer a fitness advantage, enhancing the ability of the pathogen to persist in the vagina and cause ascending infections (Kaminska et al. 2025). However, the identification of less common pilus arrangements and serotypes may indicate an ongoing adaptation of endemic strains to immune pressure, antibiotic exposure or microenvironmental differences in the genital tract, as previously suggested (Khodaei et al. 2018; Lopes et al. 2018). Alternatively, these differences could be due to the introduction into our local setting of new strains by human mobility from different geographic locations and ethnic diversities (Russell et al. 2017; Jamrozy et al. 2023).

The virulence gene repertoire identified in our collection supports our first hypothesis that traits previously associated with invasive GBS are now common in colonizing strains. Adhesion/invasion and immune evasion genes (*fbsA*, *fbsB lmb*, and *scpB*), as well as *cylB* and *hylB* were widely distributed in our isolates collection, highlighting their key role in the vaginal and rectal colonization and in pathogenicity (Rosa‐Fraile et al. 2014; Bobadilla et al. 2021; EL‐Lakany et al. 2023). Within the alpha‐like protein family, *rib* and *alpha‐C* were the most common genes in our collection. The Rib protein was predominant in serotypes III and II isolates, while alpha‐C in serotypes IV, V, and VI, supporting their contribution to host‐pathogen interactions within these lineage (EL‐Lakany et al. 2023). Regarding hemolytic phenotype, *alpha‐C* showed positive correlation (*r* = 0.54, *p* = 0.002), whereas *rib* was negatively correlated (*r* = −0.51, *p* = 0.003). This opposing pattern, together with lack of co‐occurrence (*r* = −0.60, *p* < 0.0001) (Supporting Information S1: Figure S1), suggests mutually exclusive evolutionary strategies. In addition, the association between *rib* and the PI‐1 + 2b (*r* = 0.54, *p* = 0.002) found in serotype III isolates points to a preferential linkage of virulence determinants that may synergistically promote colonization and tissue invasion (Sharma et al. 2013; Lazzarin et al. 2017; Tsai et al. 2022). Notably, isolate 10, an MDR isolate with inducible clindamycin resistance, lacked all type of alpha‐like protein genes, a rare profile that may reflect an unusual evolutionary path involving both resistance acquisition and alteration of the surface protein repertoire (Maeland et al. 2015; Bianchi‐Jassir et al. 2020). The low prevalence of *alp2/3*, the rarity of *bac* and *epsilon*, and the absence of *alp4* genes, are in line with previous studies describing the restricted distribution of some alpha‐like protein genes among particular serotypes or clonal complexes (EL‐Lakany et al. 2023; Zeng et al. 2024).

While *hvgA‐*positive strains are historically associated with neonatal meningitis, their increasing detection even in non‐pregnant adults and healthy carriers suggests silent dissemination in the community (Gong et al. 2024; Imperi et al. 2024). In our study, one‐fourth of *hvgA‐*positive strains were recovered from urine (25%, 9/12) indicating their expansion beyond the vaginal niche and supporting the notion that urinary and fecal specimens can be additional reservoirs of hypervirulent GBS, both in symptomatic infections and asymptomatic carriage (WHO 2024; Abgral et al. 2025, Piccinelli et al. 2015; Motallebirad et al. 2020). Furthermore, *hvgA*‐positive strains were strongly associated with serotype III (*r* = 0.66, *p* < 0.0001), and with pilus island combinations PI‐2b (*r* = 0.41, *p* = 0.021) and PI‐1 + 2b (*r* = 0.74, *p* < 0.0001) (Supporting Information S1: Figure S1), mirroring the well‐described serotype III‐PI‐2b combination (Hsu et al. 2023; Imperi et al. 2024). The *hvgA*‐positive HLGR strains with the serotype IV/PI‐2b/*alpha‐C* virulence profile (17%) resemble the emergent hypervirulent clone currently expanding in Italian healthcare settings (Creti et al. 2024). The presence of PI‐2b within this lineage may increase the risk of epithelial colonization and central nervous system invasion (Kaminska et al. 2025). Altogether, this overlap between enhanced virulence traits, resistance determinants and maternal colonization fits with the emerging descriptions of “dual‐threat” ST17‐related clones and reinforces the need for both rigorous antibiotic stewardship and alternative preventive strategies, including vaccines and probiotic approaches. In this context, we evaluated *Lactobacillus* spp. strains for their potential antagonistic effect against representative GBS isolates from our collection. Experimental conditions were designed to mimic the vaginal niche, with high *Lactobacillus* dosage, reflecting their dominance in healthy microbiota and low GBS infection dose. Under these conditions, *Lactobacillus* spp. strains did not affect HeLa cells, whereas GBS induced detectable damage within only 3 h. This finding was further supported by microscopic evaluation, which revealed marked cytopathic effects in HeLa cells infected with GBS alone, in contrast to the preserved epithelial morphology observed in co‐culture with *Lactobacillus* spp. (Supporting Information S1: Figure S3). Probiotic strains exerted strain‐dependent growth inhibition, preservation of epithelial viability, and marked impairment of pathogen adhesion. Co‐culture experiments confirmed strong antagonistic activity of selected *Lactobacillus* strains against GBS, with mortality rates approaching 100% for most GBS isolates. However, the magnitude of inhibition varied depending on both probiotic and pathogen genotypes and phenotypes. Spearman's correlation analysis supported these findings, revealing an increased susceptibility of serotype II to probiotic activity, whereas *L. rhamnosus* showed strain‐specific inhibition pattern activity against MDR isolates (*r* = −0.37, *p* = 0.039). In contrast, inhibition by *L. plantarum* and *L. paracasei* was positively correlated with the presence of PI‐1 + 2a (*r* = 0.39, *p* = 0.035) (Supporting Information S1: Figure S2), while no significant correlation was observed for *L. reuteri*. These results underscore that GBS susceptibility to probiotic inhibition reflects a complex interplay between bacterial capsule composition, resistance profile, as well as the metabolic and antimicrobial properties of individual *Lactobacillus* species (Martín et al. 2019; Marziali et al. 2019). Thus, to overcome strain‐specific variability, a combination of multiple *Lactobacillus* strains with complementary inhibition profiles was tested under the same experimental conditions, using a panel of GBS strains representing diverse virulence and resistance profiles. The *Lactobacillus* combination most effectively reduced adhesion of non‐hemolytic, highly aggregative *rib*‐positive isolates from diverse serotype and pilus profiles, whereas protection was less pronounced against *hvgA‐*positive, MDR, inducible clindamycin resistant, or strongly hemolytic isolates. Notably, the strong hemolytic isolates lacking MDR or hypervirulent lineage markers were more susceptible than isolates combining these virulence traits, suggesting that adhesins, toxins, resistance‐associated adaptations may impact on *Lactobacillus* competition at epithelial surfaces. The enhanced performance of the *Lactobacillus* mixture likely reflects its capacity to displace GBS from epithelial binding sites and progressively overtake the occupied niche. This overtaking behavior is consistent with *Lactobacillus*‐driven acidification and lactic acid toxicity, as the co‐culture medium became markedly acidic (4.07 ± 0.21, data not showed), indicating efficient conversion of available glucose into lactic acid (Marziali et al. 2019; Abedi and Hashemi 2020). In addition to pH‐mediated inhibition, lactobacilli may protect the epithelium through competitive exclusion at epithelial binding sites, modulation of host receptors, production of inhibitory metabolites such as organic acids, and interference with early aggregation or biofilm initiation (Martín et al. 2019; Marziali et al. 2019). Several studies highlighted the potential of vaginal lactobacilli as alternative or complementary strategies to IAP by reducing GBS carriage, limiting adhesion, and producing bacteriocin‐like molecules that inhibit a broad panel of clinical isolates (Ruíz et al. 2012; Ortiz et al. 2014; Martín et al. 2019; Marziali et al. 2019). Therefore, our results align with prior findings on the efficacy of selected *Lactobacillus* spp. against GBS, and extend this knowledge by demonstrating that multi‐strain *Lactobacillus* combinations can offer a broader and more effective protection, even against emerging ST‐17 hypervirulent or MDR GBS lineages. From a translational standpoint, such combined probiotic strategies could represent a valuable alternative or complementary approach to standard antibiotic prophylaxis, particularly in settings where hypervirulent or MDR GBS lineages circulate in the maternal vaginal reservoir. However, the present study is limited by the relatively small number of isolates from a restricted geographic area, the absence of longitudinal sampling and clinical outcome data, and the use of a HeLa‐based in vitro model, which only partially recapitulates the architecture and physiology of the vaginal mucosa. Thus, although our findings should be interpreted with caution and validated in larger cohorts and more physiologically relevant experimental systems, they provide a framework for future investigations (including advanced in vitro models, omics‐based approaches, and in vivo studies) aimed at identifying high‐risk GBS lineages and evaluating targeted multi‐strain *Lactobacillus* formulations as a non‐antibiotic strategy to limit GBS colonization.

## 5 Conclusion

Overall, our data show that colonizing GBS isolates in the maternal genital tract frequently combine invasive‐like virulence repertoires with concerning resistance profiles, including HLGR, within serotype III/PI‐2b/*hvgA*‐associated lineages. Distinct capsule‐pilus‐surface protein configurations, together with variable hemolysis and aggregation, appear to underpin multiple, strain‐specific strategies for epithelial colonization and persistence. In parallel, we demonstrate that selected multi‐strain *Lactobacillus* combinations can ecologically constrain these lineages in vitro through acidification‐driven niche modification and competition for epithelial attachment. These findings support a mechanistic model in which GBS‐*Lactobacillus* interactions at epithelial surfaces are shaped by the balance between GBS virulence architectures and *Lactobacillus*‐mediated environmental remodeling, particularly lactic acid‐driven acidification. This framework provides a basis for future translational efforts aimed at identifying high‐risk GBS lineages and developing targeted multi‐strain *Lactobacillus* formulations as non‐antibiotic strategies to reduce GBS colonization.

## Author Contributions

**Astri D Tagueha:** investigation, writing – original draft, validation, visualization, writing – review and editing. **Giulia Radocchia:** investigation, validation, writing – review and editing. **Daniela Scribano:** methodology, supervision, writing – review and editing. **Matteo Lo Scalzo:** investigation, writing – review and editing. **Giacinto Panella:** investigation, writing – review and editing. **Serena Schippa:** supervision, methodology, writing – review and editing. **Giovanni Gherardi:** methodology, supervision, writing – review and editing. **Roberta Creti:** methodology, supervision, writing – review and editing. **Ilaria Marani:** methodology, writing – review and editing. **Giovanna Alfarone:** methodology, writing – review and editing. **Monica Imperi:** methodology, writing – review and editing. **Massimiliano Marazzato:** writing – review and editing, investigation. **Lucia Nencioni:** investigation, writing – review and editing. **Paola Checconi:** validation, funding acquisition, writing – review and editing. **Dolores Limongi:** validation, funding acquisition, writing – review and editing. **Anna Teresa Palamara:** supervision, writing – review and editing. **Cecilia Ambrosi:** conceptualization, writing – original draft, writing – review and editing, methodology, visualization, investigation, validation, supervision.

## Ethics Statement

The authors have nothing to report.

## Conflicts of Interest

The authors declare no conflicts of interest.

## Supporting information


**Figure S1:** Correlation matrix of phenotypes and genotypes traits.
**Figure S2:** Lactobacillus inhibition in broth co‐culture with S. agalactiae isolates displaying phenotype and genotype traits.
**Figure S3:** Representative images of HeLa cell monolayers under S. agalactiae and Lactobacilli treatments.
**Table S1:** Susceptibility profiles of S. agalactiae clinical isolates based on MIC and disk diffusion test for selected antibiotics.
**Table S2:** Concordance between disk test and resistance genes for tetracycline, erythromycin, clindamycin, and gentamicin in S. agalactiae isolates.
**Table S3:** Primer sequences used in this study.

## Data Availability

The data that support the findings of this study are available in the supporting material of this article.
