# Supplementary material for: Facing Antimicrobial Resistance and Virulence in Streptococcus agalactiae During Late Pregnancy: Evaluation of Lactobacilli as a Supportive Approach
Source: Microbiologyopen. 2026 Jun 23;15(3):e70347. doi: 10.1002/mbo3.70347 (PMC13291431; doi:10.1002/mbo3.70347)
Supplement: Supplementary file 1 — Figure S1: Correlation matrix of phenotypes and genotypes traits. Figure S2: Lactobacillus inhibition in broth co‐culture with S. agalactiae isolates displaying phenotype and genotype traits. Figure S3: Representative images of HeLa cell monolayers under S. agalactiae and Lactobacilli treatments. Table S1: Susceptibility profiles of S. agalactiae clinical isolates based on MIC and disk diffusion test for selected antibiotics. Table S2: Concordance between disk test and resistance genes for tetracycline, erythromycin, clindamycin, and gentamicin in S. agalactiae isolates. Table S3: Primer sequences used in this study. [file MBO3-15-e70347-s001.docx]

**Facing Antimicrobial Resistance and Virulence in *Streptococcus agalactiae* during Late Pregnancy: Evaluation of *Lactobacilli* as a Supportive Approach**

**Tagueha et al.**

**SUPPLEMENTARY MATERIALS**

**Table S1.** Susceptibility profiles of *S. agalactiae* clinical isolates based on MIC and disk diffusion test for selected antibiotics.

| Strain IDs | BZP | LVX | LNZ | MXF | RIM | TEC | TGC | T/S | VAN | TET | EM | CLN | GENT | Category |
| --- | --- | --- | --- | --- | --- | --- | --- | --- | --- | --- | --- | --- | --- | --- |
| 1 | 0.12 | 0.5 | ≤2 | 0.12 | 0.25 | ≤0.12 | ≤0.06 | ≤ 10 | 0.5 | 27 | 25 | 21 | 0 |  |
| 2 | ≤0.06 | 0.5 | ≤2 | 0.25 | 0.25 | ≤0.12 | ≤0.06 | ≤ 10 | 0.5 | 26 | 25 | 24 | 0 |  |
| 3 | ≤0.06 | 0.5 | ≤2 | 0.12 | 0.25 | ≤0.12 | ≤0.06 | ≤ 10 | 0.5 | 16 | 27 | 25 | 16 |  |
| 4 | ≤0.06 | 1 | ≤2 | 0.12 | ≤0.06 | ≤0.12 | ≤0.06 | ≤ 10 | 0.5 | 27 | 24 | 21 | 15 |  |
| 5 | ≤0.06 | 1 | ≤2 | 0.12 | 0.12 | ≤0.12 | ≤0.06 | ≤ 10 | 0.5 | 27 | 25 | 22 | 16 |  |
| 6 | ≤0.06 | 0.5 | ≤2 | 0.25 | ≤0.06 | ≤0.12 | ≤0.06 | ≤ 10 | 0.5 | 27 | 26 | 24 | 16 |  |
| 7 | ≤0.06 | 1 | ≤2 | 0.25 | 0.25 | ≤0.12 | ≤0.06 | ≤ 10 | 0.5 | 16 | 0 | 0 | 17 | **MDR** |
| 8 | 0.12 | 1 | ≤2 | 0.12 | 0.12 | ≤0.12 | ≤0.06 | ≤ 10 | 0.5 | 16 | 0 | 0 | 17 | **MDR** |
| 9 | 0.12 | 0.5 | ≤2 | 0.12 | 0.12 | ≤0.12 | ≤0.06 | ≤ 10 | 0.5 | 16 | 25 | 23 | 16 |  |
| 10 | ≤0.06 | 0.5 | ≤2 | 0.12 | ≤0.06 | ≤0.12 | ≤0.06 | ≤ 10 | 0.5 | 18 | 0 | 22/0* | 0 | **MDR** |
| 11 | ≤0.06 | 1 | ≤2 | 0.12 | ≤0.06 | ≤0.12 | ≤0.06 | ≤ 10 | 0.5 | 16 | 26 | 24 | 18 |  |
| 12 | 0.12 | 1 | ≤2 | 0.12 | 0.25 | ≤0.12 | ≤0.06 | ≤ 10 | 0.5 | 17 | 13 | 22 | 17 |  |
| 13 | ≤0.06 | 1 | ≤2 | 0.12 | ≤0.06 | ≤0.12 | ≤0.06 | ≤ 10 | 0.25 | 16 | 25 | 24 | 14 |  |
| 14 | ≤0.06 | 1 | ≤2 | 0.12 | 0.12 | ≤0.12 | ≤0.06 | ≤ 10 | 0.5 | 16 | 25 | 25 | 15 |  |
| 15 | ≤0.06 | 1 | ≤2 | 0.12 | ≤0.06 | ≤0.12 | ≤0.06 | ≤ 10 | 0.25 | 15 | 0 | 0 | 16 | **MDR** |
| 16 | 0.12 | 1 | ≤2 | 0.25 | 0.12 | ≤0.12 | ≤0.06 | ≤ 10 | 0.5 | 16 | 24 | 22 | 17 |  |
| 17 | 0.12 | 1 | ≤2 | 0.25 | 0.25 | ≤0.12 | ≤0.06 | ≤ 10 | 1 | 16 | 25 | 22 | 16 |  |
| 18 | 0.12 | 0.5 | ≤2 | 0.12 | 0.12 | ≤0.12 | ≤0.06 | ≤ 10 | 0.5 | 16 | 26 | 26 | 17 |  |
| 19 | ≤0.06 | 1 | ≤2 | 0.25 | 0.25 | ≤0.12 | ≤0.06 | ≤ 10 | 0.5 | 15 | 26 | 23 | 17 |  |
| 20 | ≤0.06 | 1 | ≤2 | 0.25 | ≤0.06 | ≤0.12 | ≤0.06 | ≤ 10 | 0.5 | 26 | 26 | 24 | 16 |  |
| 21 | ≤0.06 | 1 | ≤2 | 0.25 | 0.25 | ≤0.12 | ≤0.06 | ≤ 10 | 0.5 | 15 | 0 | 22/0* | 16 | **MDR** |
| 22 | ≤0.06 | 0.5 | ≤2 | 0.12 | 0.12 | ≤0.12 | ≤0.06 | ≤ 10 | 0.5 | 16 | 27 | 25 | 14 |  |
| 23 | ≤0.06 | 0.5 | ≤2 | 0.12 | 0.12 | ≤0.12 | ≤0.06 | ≤ 10 | 0.25 | 16 | 24 | 23 | 16 |  |
| 24 | ≤0.06 | 0.5 | ≤2 | 0.12 | 0.12 | ≤0.12 | ≤0.06 | ≤ 10 | 0.5 | 27 | 25 | 23 | 18 |  |
| 25 | ≤0.06 | 1 | ≤2 | 0.25 | 0.12 | ≤0.12 | ≤0.06 | ≤ 10 | 0.5 | 28 | 25 | 24 | 15 |  |
| 26 | ≤0.06 | 1 | ≤2 | 0.25 | 0.12 | ≤0.12 | ≤0.06 | ≤ 10 | 0.5 | 15 | 25 | 23 | 15 |  |
| 27 | ≤0.06 | 1 | ≤2 | 0.25 | 0.25 | ≤0.12 | ≤0.06 | ≤ 10 | 1 | 15 | 25 | 23 | 19 |  |
| 28 | ≤0.06 | ≥16 | ≤2 | 0.25 | 0.12 | ≤0.12 | ≤0.06 | ≤ 10 | 0.5 | 14 | 0 | 0 | 17 | **MDR** |
| 29 | ≤0.06 | 0.5 | ≤2 | 0.12 | ≤0.06 | ≤0.12 | ≤0.06 | ≤ 10 | 0.5 | 22 | 28 | 28 | 19 |  |
| 30 | ≤0.06 | 0.5 | ≤2 | 0.12 | ≤0.06 | ≤0.12 | ≤0.06 | ≤ 10 | 0.5 | 15 | 0 | 0 | 17 | **MDR** |
| 31 | ≤0.06 | 0.5 | ≤2 | 0.12 | 0.12 | ≤0.12 | ≤0.06 | ≤ 10 | 0.5 | 15 | 25 | 23 | 17 |  |

MICs for BZP, Benzylpenicillin (R>0.125), LVX, Levofloxacin (R>2), LNZ, Linezolid (R>2), MXF, Moxifloxacin (R>0.5), RIM, Rifampicin (R>0.06), TEC, Teicoplanin (R>2), TGC, Tigecycline (R>0.12), T/S, Trimethoprim/Sulfamethoxazole (R>20), and VAN, Vancomycin (R>2), TET, Tetracycline (R>1); EM, Erythromycin (R>0.25), and CLN, Clindamycin (R>0.5) were determined using the VITEK® 2 system and interpreted according to EUCAST clinical breakpoints (v15.0). Resistance to TET, Tetracycline (R<23), EM, Erythromycin (R=0), CLN, Clindamycin (R<20), and GENT, Gentamicin (R=0) was assessed by disk diffusion (30 µg TET, 15 µg EM, 2 µg CLN, and 500 µg GENT). Inducible clindamycin resistance was specifically detected using the D-test method. An asterisk (*) indicates inducible clindamycin resistance. MDR, multidrug resistant.

**Table S2.** Concordance between disk test and resistance genes for tetracycline, erythromycin, clindamycin, and gentamicin in *S. agalactiae* isolates.

| **Strain IDs** | **TET** | **TET**  **pheno-type** | ***tetM* gene** | ***tetO* gene** | **EM** | **CLN** | **CR/IR/M pheno-type** | ***ermA* gene** | ***mef* gene** | ***ermB* gene** | **GENT** | **HLGR** | ***aac(6')-Ie-aph(2")-Ia* gene** |
| --- | --- | --- | --- | --- | --- | --- | --- | --- | --- | --- | --- | --- | --- |
| 1 | 27 | S | - | - | 25 | 21 | S | - | - | - | 0 | R | + |
| 2 | 26 | S | - | - | 25 | 24 | S | - | - | - | 0 | R | + |
| 3 | 16 | R | + | - | 27 | 25 | S | - | - | - | 16 | S | - |
| 4 | 27 | S | - | - | 24 | 21 | S | - | - | - | 15 | S | - |
| 5 | 27 | S | - | - | 25 | 22 | S | - | - | - | 16 | S | - |
| 6 | 27 | S | - | - | 26 | 24 | S | - | - | - | 16 | S | - |
| 7 | 16 | R | + | - | 0 | 0 | CR | - | - | + | 17 | S | - |
| 8 | 16 | R | + | - | 0 | 0 | CR | - | - | + | 17 | S | - |
| 9 | 16 | R | + | - | 25 | 23 | S | - | - | - | 16 | S | - |
| 10 | 18 | R | + | - | 0 | 22/0* | IR | - | - | - | 0 | R | + |
| 11 | 16 | R | + | - | 26 | 24 | S | - | - | - | 18 | S | - |
| 12 | 17 | R | + | - | 13 | 22 | M | - | + | - | 17 | S | - |
| 13 | 16 | R | - | + | 25 | 24 | S | - | - | - | 14 | S | - |
| 14 | 16 | R | + | - | 25 | 25 | S | - | - | - | 15 | S | - |
| 15 | 15 | R | - | + | 0 | 0 | CR | - | - | + | 16 | S | - |
| 16 | 16 | R | + | - | 24 | 22 | S | - | - | - | 17 | S | - |
| 17 | 16 | R | + | - | 25 | 22 | S | - | - | - | 16 | S | - |
| 18 | 16 | R | + | - | 26 | 26 | S | - | - | - | 17 | S | - |
| 19 | 15 | R | + | - | 26 | 23 | S | - | - | - | 17 | S | - |
| 20 | 26 | S | - | - | 26 | 24 | S | - | - | - | 16 | S | - |
| 21 | 15 | R | + | - | 0 | 22/0* | IR | + | - | - | 16 | S | - |
| 22 | 16 | R | - | + | 27 | 25 | S | - | - | - | 14 | S | - |
| 23 | 16 | R | + | - | 24 | 23 | S | - | - | - | 16 | S | - |
| 24 | 27 | S | - | - | 25 | 23 | S | - | - | - | 18 | S | - |
| 25 | 28 | S | - | - | 25 | 24 | S | - | - | - | 15 | S | - |
| 26 | 15 | R | - | + | 25 | 23 | S | - | - | - | 15 | S | - |
| 27 | 15 | R | + | - | 25 | 23 | S | - | - | - | 19 | S | - |
| 28 | 14 | R | + | - | 0 | 0 | CR | - | - | + | 17 | S | - |
| 29 | 22 | R | + | - | 28 | 28 | S | - | - | - | 19 | S | - |
| 30 | 15 | R | + | - | 0 | 0 | CR | - | - | + | 17 | S | - |
| 31 | 15 | R | + | - | 25 | 23 | S | - | - | - | 17 | S | - |

Tetracycline (TET), Erythromycin (EM), Clindamycin (CLN), and GENT (Gentamycin) phenotype were assessed by disk diffusion test. CR, clindamycin resistance (CLN‑R/EM‑R); IR, inducible clindamycin resistance (EM‑R/CLN‑S with D‑shaped inhibition); M, M‑phenotype (EM‑R/CLN‑S without flattening); R, resistant; and S, susceptible. HLGR was defined by reduced inhibition around high‑content gentamicin and correlated with *aac(6')-Ie-aph(2")-Ia*. The D‑test phenotypes showed the consistent presence of macrolide–lincosamide resistance genes (*ermA, ermB, mef*), except for strain 10.

**Table S3.** Primer sequences used in this study.

| *Category* | *Genes* | Primers (5’-3’) | References |
| --- | --- | --- | --- |
| Serotypes | cpsI-Ia-6-7-F | GAATTGATAACTTTTGTGGATTGCGATGA | (Imperi *et al.*, 2010) |
|  | cpsI-6-R | CAATTCTGTCGGACTATCCTGATG |  |
|  | cpsI-7-R | TGTCGCTTCCACACTGAGTGTTGA |  |
|  | cpsI-7-9-F | CTGTAATTGGAGGAATGTGGATCG |  |
|  | cpsI-9-R | AATCATCTTCATAATTTATCTCCCATT |  |
|  | cpsL-F | CAATCCTAAGTATTTTCGGTTCATT |  |
|  | cpsL-R | TAGGAACATGTTCATTAACATAGC |  |
|  | cpsG-F | ACATGAACAGCAGTTCAACCGT |  |
|  | CpsG-R | ATGCTCTCCAAACTGTTCTTGT |  |
|  | CpsG-2-3-6-R | TCCATCTACATCTTCAATCCAAGC |  |
|  | CpsN-5-F | ATGCAACCAAGTGATTATCATGTA |  |
|  | CpsN-5-R | CTCTTCACTCTTTAGTGTAGGTAT |  |
|  | CpsJ-8-F | TATTTGGGAGGTAATCAAGAGACA |  |
|  | CpsJ-8-R | GTTTGGAGCATTCAAGATAACTCT |  |
|  | cpsJ-2-4-F | CATTTATTGATTCAGACGATTACATTGA |  |
|  | cpsJ-2-R | CCTCTTTCTCTAAAATATTCCAACC |  |
|  | cpsJ-4-R | CCTCAGGATATTTACGAATTCTGTA |  |
|  | cpsJ-Ib-F | GCAATTCTTAACAGAATATTCAGTTG |  |
|  | cpsJ-Ib-R | GCGTTTCTTTATCACATACTCTTG |  |
| Pilus Island | adhP_F162 | ACGCATTTTGGGTCACGA | (Springman *et al.*, 2014) |
|  | adhP_R944 | GTATCCACAGGCACTTTTTCAAC |  |
|  | SAG647_F496 | CTACCAACGGCCAAGCTATTTACC |  |
|  | SAG647_R889 | TAGCCGCTTTTTCATTCTTTCTCC |  |
|  | SAG1406_F356 | AACTCCCTATATTTGCAGGTTCAA |  |
|  | SAG1406_R598 | CGGGTGTAACGACTTTTATCTGAT |  |
|  | SAN1517_F57 | GGGGGTAGGCTTAATGGCTTAT |  |
|  | SAN1517_R575 | TCCGGTTTAACTGTTCTGATTTGAT |  |
| Virulence genes | *fbsA* | F-TGTAGCTAATGGACCGATGTT  R-TTTTCATTGCGTCTCAAACC | (Bobadilla *et al.*, 2021) |
|  | *fbsA* | F-TGTAGCTAATGGACCGATGTT  R-TTTTCATTGCGTCTCAAACC |  |
|  | *fbsB* | F-ACAACTGCGGAAATGACCTC  R-ACGAGCGACGTTGAATTCTT |  |
|  | *lmb* | F-GACGCAACACACGGCAT  R-TGATAGAGCACTTCCAAATTTG |  |
|  | *cylB* | F-GGGCTGCAGGTATTATCGAA  R-ATTTCCACCAAAAGCAAACG |  |
|  | *hylB* | F-TTATCATCCAGCGCCTCCTAG  R-GTGGTGATAACTGACTTCTTGGGA |  |
|  | *scpB* | F-AGCCATATGCTGCGATCTCT  R-GGGTTGAACCAAGTGTGCTT |  |
|  | *bac* | F-TGTAAAGGACGATAGTGTGAAGAC  R-CATTTGTGATTCCCTTTTGC |  |
|  | *rib* | F-CAGGAAGTGCTGTTACGTTAAAC  R-CGTCCCATTTAGGGTCTTCC |  |
|  | *hvgA*_(ST-17S) | ATACAAATTCTGCTGACTACCG | (Lamy *et al.*, 2006) |
|  | hvgA_ST-17AS | TTAAATCCTTCCTGACCATTCC |  |
|  | Universal-F | TGATACTTCACAGACGAAACAACG | (Creti *et al.*, 2004) |
|  | *AlphaC*-R | TACATGTGGTAGTCCATCTTCACC |  |
|  | *Epsilon*-R | CCAGATACATTTTTTACTAAAGCGG |  |
|  | *Alp2/3*-R | CACTCGGATTACTATAATATTTAGCAC |  |
|  | *Alp4*-R | TTAATTTGCACCGGATTAACACCAC |  |


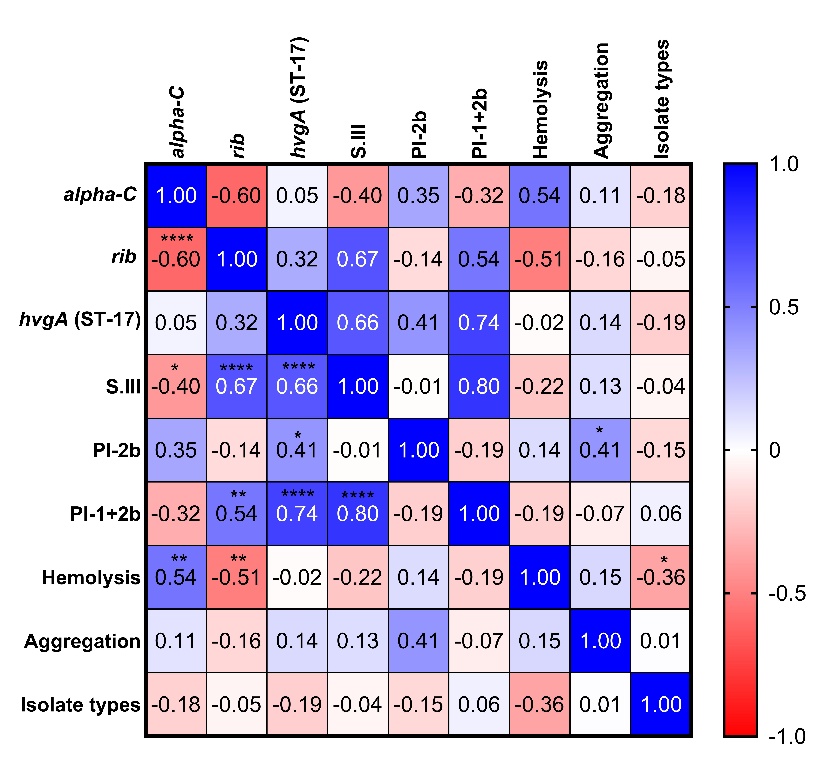


**Figure S1. Correlation matrix of phenotypes and genotypes traits.** Parameters included in the analysis are reported. Data were analyzed using Spearman’s correlation. The colors represent correlation coefficient; its intensity depicts the coefficient’s value (shades of blue are positive correlations and shades of red are negative correlations). The graph was generated using GraphPad Prism version 11. * P < 0.05, ** P < 0.01, ****P* < 0.001, *****P* < 0.0001.


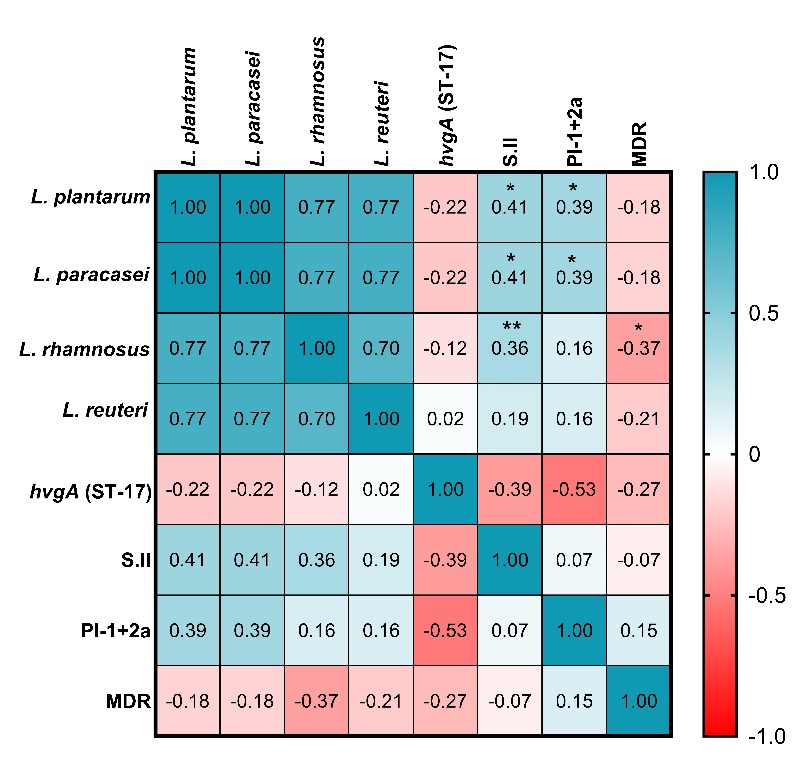


**Figure S2. *Lactobacillus* inhibition in broth co-culture with *S. agalactiae* isolates displaying phenotype and genotype traits**. Parameters included in the analysis are reported. Data were analyzed using Spearman’s correlation. The colors represent correlation coefficient; its intensity depicts the coefficient’s value (shades of green are positive correlations and shades of red are negative correlations). The graph was generated using GraphPad Prism version 11. *P < 0.05, ** P < 0.01.


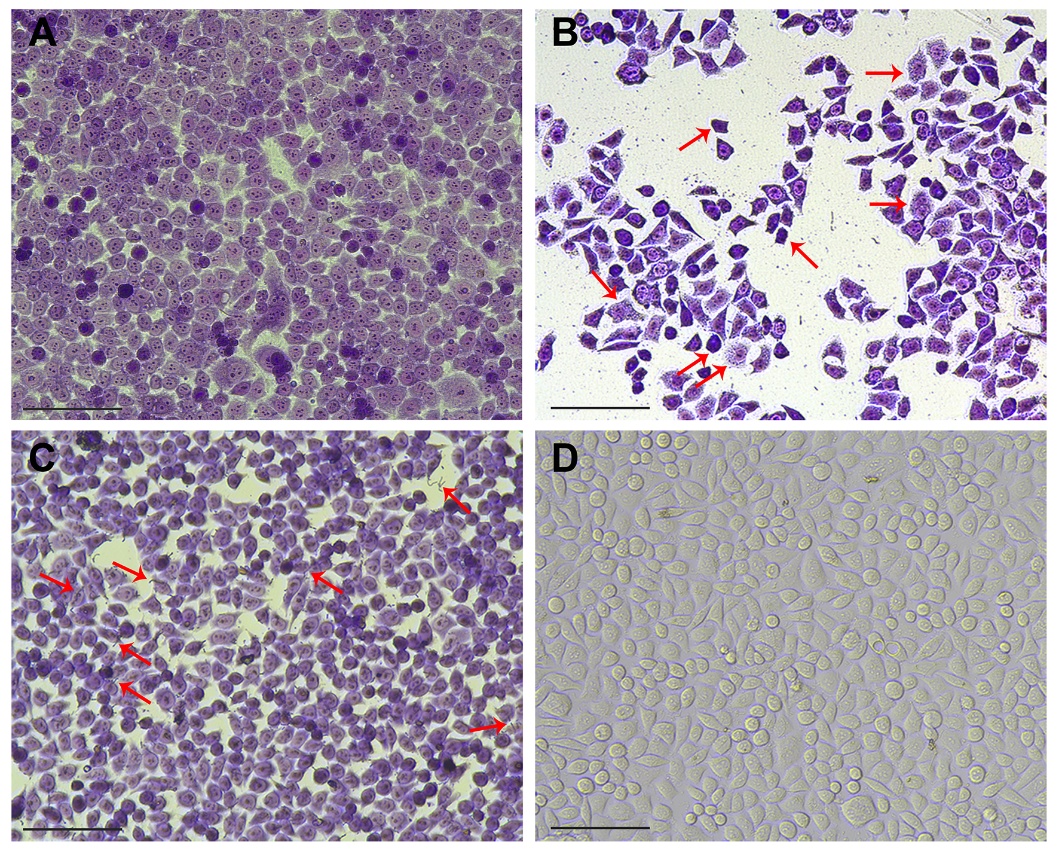


**Figure S3.** **Representative images of** **HeLa cell monolayers under *S. agalactiae* and *Lactobacilli* treatments.** (A) Uninfected HeLa cell monolayer. (B) HeLa cells infected with *S. agalactiae*; the red arrow points to a dying cell with cytopathic changes. (C) HeLa cells co-infected with *S. agalactiae* and the *Lactobacillus* combination; the red arrow points to *Lactobacillus* cells. (D) Phase-contrast image of HeLa cells treated with the *Lactobacillus* combination after five washing steps before the MTT assay.
